# Counterspeech encouraging users to adopt the perspective of minority groups reduces hate speech and its amplification on social media

**DOI:** 10.1038/s41598-025-05041-w

**Published:** 2025-07-01

**Authors:** Gloria Gennaro, Laurenz Derksen, Aya Abdelrahman, Emma Broggini, Mariya Alexandra Green, Victoria Andrea Haerter, Elia Heer, Isabel Heidler, Fiona Kauer, Han-Nuri Kim, Benjamin Landry, Alessio Levis, Jiazhen Li, Şevval Şimşir, Iva Srbinovska, Robin Anna Vital, Karsten Donnay, Fabrizio Gilardi, Dominik Hangartner

**Affiliations:** 1 Department of Political Science, University College London, London, WC1H9QU UK; 2 Center for Comparative and International Studies, ETH Zurich, 8092 Zurich, Switzerland; 3 Department of Political Science, University of Zurich, 8050 Zurich, Switzerland; 4 Department of Social Sciences, University of Bern, 3012 Bern, Switzerland; 5 School of Business and Economics, University of Tübingen, 72074 Tübingen, Germany

**Keywords:** Hate speech, Social media, Counterspeech, Field experiment, Psychology, Human behaviour

## Abstract

Online intergroup hostility is a pervasive and troubling issue, yet experimental evidence on how to curb it remains scarce. This study examines counterspeech as a user-driven strategy to reduce hate speech. Drawing on theories that suggest adopting the perspective of minority groups can reduce prejudice, we randomized four counterspeech strategies across the senders of 2102 xenophobic Twitter messages. Compared to a passive control group, we find that the pooled effect of the three perspective-centered strategies—traditional perspective-taking, analogical perspective-taking, and perspective getting—increased the likelihood that the sender deleted their xenophobic message by +0.14 SD ($p=0.003$), decreased the number of likes the xenophobic message received by others (− 0.133 SD, $p=0.040$), but yielded a limited and not statistically significant estimate for the share of xenophobic messages the sender posted over the following four weeks (− 0.084 SD, $p=0.178$). Differences between the three perspective-centered strategies were generally small and not statistically significant, though analogical perspective-taking—encouraging senders to compare their own experiences of being attacked online with their discriminatory behavior toward outgroups—appears to have slightly larger effects across multiple outcomes. Disapproval messages without a perspective shift produced smaller and non-significant estimates. These findings advance our theoretical understanding of how counterspeech works and provide actionable insights for how users can contribute to reducing intergroup hostility and its amplification online—especially at a time when many platforms are scaling back content moderation.

## Introduction

Social interactions increasingly take place online. This trend offers exciting opportunities for exchange beyond traditional community boundaries, but it also raises significant challenges. Among those, the rise of online hate speech has spurred important debates on the governance of online speech^1^. Elon Musk’s acquisition of Twitter has recently put those challenges back in the spotlight of regulators^2^. While hate speech—hostile language targeted against a person or group because of their actual or perceived innate characteristics^3^—is not new, the internet has amplified its reach. The consequences for individuals targeted by hate speech can be severe. Research shows that the emotional and physiological reactions to online hostility and harassment are similar to offline harassment^4^, and hateful social media posts can propagate real-life violence^5^. These harms disproportionately affect minorities, who are more likely to be targeted by hate speech, creating disparities in the costs and benefits of engaging in online public discourse.

Recent advances in social psychology and political science have focused on three promising strategies for reducing intergroup hostility in offline settings—all aimed at fostering empathy and understanding among majority group members toward minorities^6,7^. The first approach, called *traditional perspective-taking*, involves individuals assuming the perspective of outgroup members^8^. Successful interventions in this area have used immersive exercises that require individuals to imagine and describe the experiences of outgroup members^8,9^. The second approach, known as *analogical perspective-taking*, prompts individuals to draw analogies between their own experiences of hostility or discrimination and those of outgroup members^6,10^. The third approach, called *perspective-getting*, provides individuals with direct testimonies or stories from outgroup members, either through personal accounts or narratives shared by ingroup members^7,11^.

**Fig. 1:**
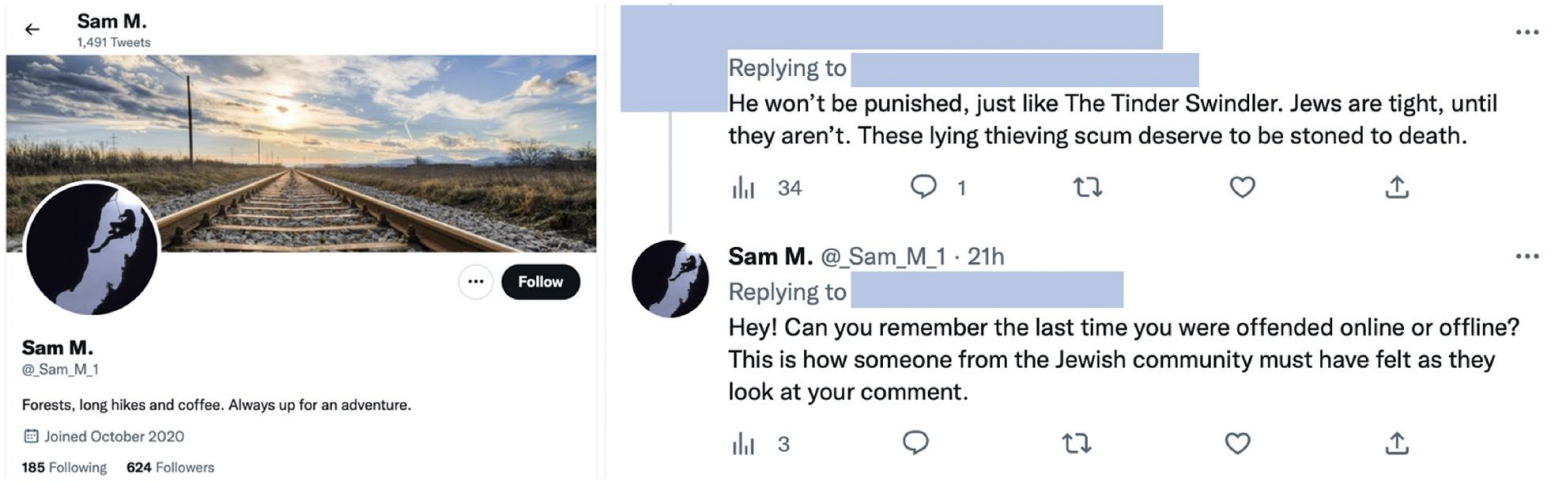
Example of a human-controlled sock-puppet account (left) and counterspeech message (right). Identifying information is obscured to protect the sender’s anonymity.

Building upon these findings, our research aims to investigate whether these approaches can effectively reduce the spread of online hate speech. We conducted a social media field experiment utilizing counterspeech strategies in a real-world online setting. In our preregistered experiment, counterspeech was deployed to respond to 2102 racist or xenophobic tweets from Twitter users based in the United States. The intervention involved delivering a direct and publicly visible counterspeech response to the xenophobic tweet within 24 h. To administer the treatments, we utilized human-controlled sockpuppet accounts. Fig. 1 provides an example of a sockpuppet account and a counterspeech message.

Study subjects were randomly assigned to one of four treatment groups or a control group, with assignment probabilities held constant across the study period. Three treatments implemented the strategies designed to encourage users to adopt the perspective of minority groups—traditional perspective-taking, analogical perspective-taking, and perspective getting—discussed above. The fourth treatment used a disapproval message^12^. While such messages are widely used in practice^13,14^, evidence of their effectiveness in experimental settings is mixed^15,16^. We included the disapproval message as a benchmark for non-perspective-centered counterspeech, rather than using a neutral engagement message as an active control, given concerns that neutral replies may inadvertently boost the visibility and amplification of hate content.

Previous experimental research has primarily focused on the role of the counterspeaker and how their status, legitimacy, and identity can moderate the hostile behavior of treated subjects^15,17,18^. The study most closely related to ours is that of Hangartner et al. (2021), who identified empathy-based counterspeech as particularly promising among a set of commonly used strategies. They found that responding to xenophobic tweets with perspective-getting messages—in which ingroup members relay the experiences of outgroup individuals—significantly increased tweet deletions by the original senders^19^.

Building upon this literature, our study systematically compares different perspective-centered strategies rooted in social psychology theory. We break new ground by showing that counterspeech can influence not only the sender of hate speech but also bystanders—users who engage with the xenophobic message by liking or sharing it. Recent studies highlight the important role of bystanders in the context of offline hate crimes^20,21^. We explore the possibility that counterspeech affects the senders of xenophobic messages directly, through persuasion, and indirectly, by discouraging bystander engagement and depriving senders of positive reinforcement. In the context of social media, where algorithmic propagation and amplification drive visibility, these indirect effects on bystanders may be as consequential as the direct effects on the sender.

## Results

For each outcome, we report the group mean and confidence intervals, and results from a preregistered ordinary least squares regression of each treatment against the control group (without adjusting for covariates). As expected given that the treatments were randomized, results from a second preregistered specification with covariates are virtually identical, and reported in Tables s1 and s2. In addition, in an exploratory analysis, we compare the effects of individual perspective-centered treatments against the disapproval condition. To assess statistical significance, we use two-sided tests and *p*-values $\le 0.05$. For pre-registered analysis, we report Benjamini-Hochberg $p_{BH}$-values that account for multiple hypothesis testing in addition to standard *p*-values; c.f. Methods for details. SI Section 2 reports all deviations from the pre-analysis plan.

### Effects of counterspeech interventions on xenophobic speech

**Fig. 2:**
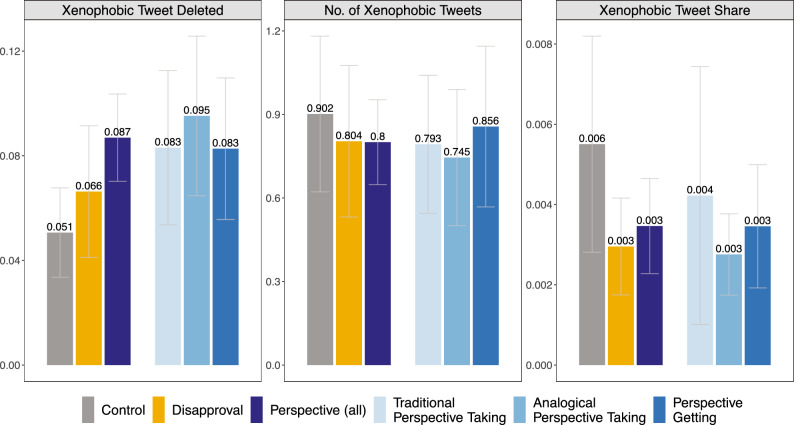
The effect of counterspeech on the sender’s behavior. Vertical bars are group-level averages along 95% confidence intervals measuring the propensity that the user deleted the original xenophobic tweet (*Xenophobic Tweet Deleted*), the number of xenophobic tweets (*No. of Xenophobic Tweets*), and the share of xenophobic to total tweets (*Xenophobic Tweet Share*), across the different treatment and control groups over the 4-week follow-up period.

The first panel in Fig. 2 shows the proportion of users who deleted their xenophobic tweets. This share increases from 5.1% in the control group (grey bar, first from the left) to 6.6% (yellow bar, second from the left) in the disapproval condition and 8.7% (dark-blue bar, third from the left) for users who received any of the perspective-centered treatments. Linear regression estimates show that the pooled effect of the perspective-centered interventions is statistically significant, increasing tweet deletion by $\beta =0.140$ standard deviations compared to the control group ($SE=0.047$, $p=0.003$, $p_{BH}=0.012$). In contrast, the disapproval messages lead to a smaller and non-significant estimate ($\beta = 0.061$, $SE=0.060$, $p=0.313$, $p_{BH}=0.626$). Next, we explore how counterspeech affects the share of xenophobic tweets in the four weeks following the intervention. In the control group, xenophobic tweets account for 0.6% of total tweets, compared to 0.3% among users in the disapproval-based treatment ($\beta = -0.105$, $SE = 0.062$, $p = 0.093$, $p_{BH} = 0.372$) and those in the perspective-centered treatments ($\beta = -0.084$, $SE = 0.062$, $p = 0.178$, $p_{BH} = 0.356$). While these estimates are directionally consistent with a reduction, they are not statistically significant. In line with these results, we also observe small and negative but statistically non-significant estimates for the effect of counterspeech interventions on the absolute number of xenophobic tweets sent. Similarly, we find limited evidence that our counterspeech messages affect the total number of tweets sent during the follow-up period (see SI Section 1).

Our multi-arm experimental design allows assessing the effectiveness of different perspective-centered strategies. The three light blue bars on the right of each panel in Fig. 2 distinguish the effects of traditional perspective-taking, analogical perspective-taking, and perspective-getting. Compared to the control group, analogical perspective-taking increases the propensity of deletion by $\beta =0.172$ SD ($SE=0.069$, $p=0.013$, $p_{BH}=0.052$), while the corresponding estimate is smaller and not statistically significant for traditional perspective-taking ($\beta =0.125$, $SE=0.067$, $p=0.063$, $p_{BH} = 0.252$) and only the unadjusted estimate is significant for perspective-getting ($\beta =0.124$, $SE=0.063$, $p=0.050$, $p_{BH}=0.200$). Counterspeech encouraging analogical perspective-taking also reduces the *Xenophobic Tweet Share* in the following four weeks by $\beta =-0.113$ standard deviations relative to the control group, a difference that is, however, not statistically significant at the 5% level ($SE=0.061$, $p=0.064$, $p_{BH}=0.128$). Again, interventions based on traditional perspective-taking ($\beta =-0.053$, $SE=0.089$, $p=0.554$, $p_{BH}=0.760$) and perspective-getting ($\beta =-0.084$, $SE=0.066$, $p=0.200$, $p_{BH}=0.400$) generate smaller and non-significant estimates. When interpreting the relative effectiveness of the three perspective-centered interventions, it is important to keep in mind that given our sample size, comparison across those may suffer from low power to detect small differences. Contrary to our preregistered hypothesis, we find that all three perspective-centered strategies have qualitatively similar effects: While analogical perspective-taking tends to perform slightly better across multiple outcomes, the differences to traditional perspective-taking and perspective-getting are not statistically significant. Seemingly unrelated regression (SUR) model, based on a sample restricted to observations with non-missing outcomes, allow us to conduct joint significant tests across all outcomes. Preregistered *F*-tests reject the joint null hypothesis of no effect across all three outcomes for the pooled perspective-centered treatments ($p=0.031$) and analogical perspective-taking ($p=0.031$), but not for traditional perspective-taking ($p=0.423$), perspective-getting ($p=0.169$), or disapproval ($p=0.389$).

We also evaluate the effectiveness of perspective-centered counterspeech strategies against the disapproval condition. Among all perspective treatments, we find for analogical perspective-taking the largest increase in xenophobic tweet deletion—$\beta _L=0.133$ SD ($SE=0.081$, $p=0.100$) compared to the disapproval group—but note that this difference is not significant at the 5% level. When comparing traditional perspective-taking and perspective getting, respectively, to disapproval, the differences are more muted and not statistically significant (traditional perspective-taking: $\beta _L=0.068$, $SE=0.077$, $p=0.382$; perspective getting: $\beta _L=0.071$, $SE=0.076$, $p=0.350$).

### Effects of counterspeech interventions on the amplification of xenophobic tweets

Next, we explore whether counterspeech can influence how other social media users engage with the xenophobic tweet. In particular, we look at the *No. of likes received by xenophobic tweet*, and the *No. of retweets of xenophobic tweet* of the original hate tweet, during the four weeks following the intervention. This analysis is in part exploratory, since we preregistered the first but not the second outcome. Likes and retweets imply that other social media users (i.e., bystanders) amplify the xenophobic tweet. In line with this interpretation, both outcomes take the value 0 for users who have deleted their xenophobic tweets, so further engagement is not possible. We expect that counterspeech may act partially as social sanctioning against norm violators, hence reducing the engagement of bystanders with the original hate tweet. However, we had no prior expectations about how the different counterspeech strategies might differ in their impact on amplification.

**Fig. 3:**
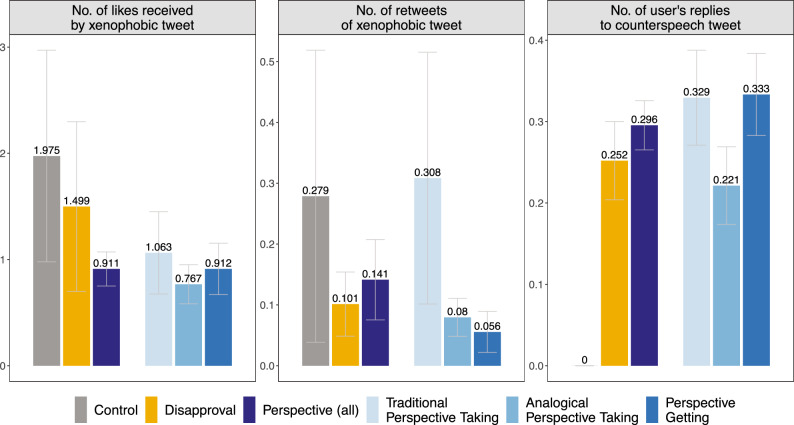
Counterspeech and bystanders’ engagement. Vertical bars are group-level averages along 95% confidence intervals measuring the number of likes received by the xenophobic tweet (*No. of likes received by xenophobic tweet*), the number of retweets of the xenophobic tweet (*No. of retweets of xenophobic tweet*), and the number of user’s replies in the same thread (*No. of user’s replies to counterspeech tweet*), across the different treatment and control groups over the 4-week follow-up period.

The first panel of Fig. 3 shows that the xenophobic tweets in the control group receive, on average, 1.975 likes. The number of likes decreases to 1.499 in the disapproval condition and to 0.911 in the pooled perspective condition. Regression estimates show that xenophobic tweets in the perspective-centered treatment arm receive $\beta =-0.133$ standard deviations fewer likes than those in the control group ($SE=0.064$, $p=0.040$, $p_{BH}=0.080$). This difference is smaller and not statistically significant for disapproval messages ($\beta =-0.059$, $SE=0.082$, $p=0.467$, $p_{BH}=0.467$). While all the different perspective-centered treatments tend to reduce the number of likes (Traditional perspective taking: $\beta =-0.114$, $SE=0.068$, $p=0.096$, $p_{BH}= 0.192$. Perspective getting: $\beta =-0.132$, $SE=0.065$, $p=0.043$, $p_{BH}= 0.073$ ), analogical perspective-taking leads, again, to the largest effect with the smallest *p*-value compared to the control condition ($\beta =-0.150$, $SE=0.065$, $p=0.020$, $p_{BH}=0.040$). When comparing analogical perspective-taking to disapproval, the difference is not significant at 5% level ($\beta _L=-0.092$, $SE=0.054$, $p=0.091$). While the direction of the estimates are the same for the two other perspective-centered interventions, they do not significantly differ from disapproval.

The second panel of Fig. 3 shows a similar pattern for the *No. of retweets of xenophobic tweet*. On average, xenophobic tweets in the control group receive 0.279 retweets. This number decreases to 0.101 retweets in the disapproval condition and to 0.141 retweets for the pooled perspective condition. The differences between the counterspeech interventions and the control group are, however, not statistically significant (Pooled perspective condition: $\beta =-0.073$, $SE=0.068$, $p=0.282$, $p_{BH}=0.282$; disapproval: $\beta =-0.094$, $SE=0.067$, $p=0.159$, $p_{BH}=0.318$). Among the different perspective-centered treatments, tweets countered with perspective-getting receive the lowest number of retweets, but these estimates are not significant at the 5% level, both against the control group ($\beta = -0.118$, $SE=0.066$, $p=0.073$, $p_{BH}=0.073$) and the disapproval condition ($\beta _L=-0.030$, $SE=0.016$, $p=0.065$). The difference between analogical perspective-taking and the control group is slightly smaller and not statistically significant ($\beta = -0.106$, $SE=0.066$, $p=0.109$, $p_{BH}=0.109$). Conversely, hate tweets receive, if anything, more retweets in the traditional perspective-taking condition compared to the disapproval condition ($\beta _L=+0.098$, $SE=0.058$, $p=0.090$), but, again, these differences are not significant at the 5% level.

To capture a third dimension of engagement, the right panel of Fig. 3 analyzes whether and how often the sender of the hate speech tweet replied to the counterspeech message (this outcome is naturally 0 for the control group). Focusing on our preregistered outcome capturing the total *No. of users’ replies to counterspeech tweet*, we find that counterspeech messages employing perspective-getting are slightly more likely to receive replies than interventions based on disapproval ($\beta _L = 0.159$, $SE=0.091$, $p=0.080$), while counterspeech messages employing analogical perspective-taking are slightly less likely to invite a reply ($\beta _L = -0.145$, $SE=0.086$, $p=0.093$)—note, however, that those differences are not statistically significant at the 5% level. Counterspeech messages employing traditional perspective-taking do not significantly differ from disapproval messages in their likelihood to receive a reply ($\beta _L = -0.064$, $SE=0.095$, $p=0.497$). Two members of the research team independently coded the $N=418$ replies and resolved the few disagreements through discussion. Their exploratory analysis reveals that $N=13$ responses are apologetic, $N=180$ double down on the xenophobic message or attack the sockpuppet account, and $N=225$ are neutral. We find no evidence that one of the counterspeech strategies invites more backlash or attacks (see Table s3). The SI Section 1.3 reports similar results from robustness tests that transform the two amplification measures to minimize the influence of outliers.

## Discussion

This study shows that addressing online hate speech with counterspeech messages can be an effective way to induce a change in the sender’s behavior. This bolsters confidence in a growing body of research that identifies counterspeech as a liberal, bottom-up complement to more restrictive content moderation policies^19^. In addition, we provide new evidence that counterspeech can affect bystanders’ expressions of support for existing hate tweets, such as likes and retweets. This suggests that counterspeech may help reduce not only the prevalence of hate speech, but also its visibility and amplification online.

Our findings on the relative impact of different counterspeech strategies are more muted, as the differences between analogical perspective-taking and the other two perspective-centered interventions are generally not statistically significant. Yet, contrary to our preregistered hypothesis, we do not find that perspective getting yields consistently larger effects across outcomes. If anything, analogical perspective-taking appears to perform slightly better than traditional perspective-taking and perspective-getting, although the differences are small and not statistically significant. Clearly, more research with larger samples and statistical power is needed to confirm this pattern.

If replicated, these findings would stand in contrast to a previous study^7^ that found perspective-getting narratives—when embedded in face-to-face conversations—to be a main driver of reduced intergroup prejudice. A plausible explanation for this potential discrepancy lies in the different constraints imposed by offline and online interventions^22^. The offline intervention examined by the previous study^7^ involves in-depth conversations between experimenters and participants. In contrast, our one-shot interactions are constrained by the limited space (and depth) of social media posts. These contextual differences may moderate the relative effectiveness of the three perspective-centered strategies. Perspective-focused interventions are particularly effective when they “transport” the receiver into the story of a victimized group member^23^. Since this mechanism often works through the recalling of self- or other-relevant memories^24^, analogical perspective-taking may be more powerful in constrained online settings, as it explicitly draws on experiences that are already familiar to the message recipient.

Online interventions on social media also need to consider that the mechanisms through which the effect of counterspeech travels in such contexts may differ from those in offline interventions. While a previous study with face-to-face interactions^7^ documents a reduction in hostile *attitudes*, our study primarily focuses on the public *expression* of hostility on social media. The public nature of both treatment and outcome, and the presence of bystanders who can react to either, suggest that multiple mechanisms may be at play. On the one hand, senders of hate speech may be persuaded by the message and change their attitudes toward minorities, as in prior work^7^. On the other, senders may alter their behavior in response to social sanctioning, manifested as disengagement with their post. This disengagement deprives the sender of positive feedback and may reduce the likelihood of future moral transgressions^25^. The negative effect of all counterspeech strategies on bystanders’ engagement is consistent with both mechanisms contributing to behavior change. Given that analogical perspective-taking yielded slightly stronger effects than the other perspective-centered strategies, a key question for future research is whether it works by changing private attitudes or by reducing willingness to express hostility publicly.

We acknowledge that our study captures a specific moment on a constantly evolving platform. While Twitter (now X) has since undergone many changes, our findings remain relevant as they highlight scalable, user-driven interventions at a time when platform-led content moderation is being rolled back and online hate is on the rise. They also have direct implications for governments, NGOs, and social media companies committed to reducing hate speech. In a context where content moderation is increasingly contested^26^, our study underscores the potential of counterspeech as a liberal, non-coercive complement to more restrictive approaches—such as removing harmful content or banning users who post it.

## Methods

This study was approved by the ETH Zurich Ethics Committee (2021-N-178) and preregistered at https://osf.io/k4mrw.

### Sampling

The field experiment took place from November 17, 2021, to January 30, 2022. We gathered tweets in English that contained one or multiple words from a dictionary of xenophobic terms and racial slurs, and neutral terms referring to commonly targeted groups (e.g., “migrants,” “Muslims,” or “Jews”). The dictionary is reported in the Supplementary Information (SI). To narrow down this initial sample, we firstly excluded all verified user accounts, because those mainly consist of corporate accounts, organizations, governments, celebrities, or journalists. Additionally, we excluded accounts with fewer than five followers, as likely bots. We removed retweets and tweets in which the targeted group only appeared in a quote, to focus on the creation of hate speech rather than its dissemination. Furthermore, we excluded tweets that appeared humorous or sarcastic, or likely generated by bots or minors. Our analysis primarily focused on Twitter users likely based in the United States (see SI for details).

Next, we manually reviewed each tweet to determine if it contained xenophobic hate speech. To aid in this task, we ranked the tweets based on the predicted probability of containing xenophobic speech. These predicted probabilities were generated by a deep-learning classifier trained on tweets from a related study that employed the same definition of hate speech^19^. To be classified as hate speech, tweets had to meet the following criteria: (i) they included explicit references to group identifiers, such as xenophobic or racist slurs, or neutral terms; (ii) they attacked, used pejorative language, or employed discriminatory language against individuals or groups based on their (perceived) religion, ethnicity, nationality, race, color, or descent. Following this sampling procedure, we obtained a total sample of 2441 tweets and their senders, which closely approached the target size specified in the preregistered power analysis (2460).

### Experimental design

We employed random assignment to assign study subjects to one of four treatment arms, each with a 17.5% probability, or the control group (which did not receive any counter-speech) with a 30% probability. In the *traditional perspective-taking* condition, the message was designed to prompt subjects to contemplate the hurtful impact of reading their xenophobic message. For example: “Consider how hurtful it would be if you were an [Asian-American] and read this... .” In the *analogical perspective-taking* condition, subjects were encouraged to recall a personal situation in which they were attacked and draw an analogy with the experience of the person they targeted. An example message would be: “Think about the last time someone attacked you on Twitter. This is how a member of the [Asian-American community] must feel when reading your comment....” In the *perspective-getting* condition, users were provided with the perspective of a member of the targeted outgroup through the words of a potentially ingroup friend or colleague. An example message would be: “My [Asian-American] friend/colleague/coworker has received Tweets like this; they hurt every time.” To establish a benchmark with other interventions, we included a fourth condition utilizing *disapproval*. The message expressed dissatisfaction and indicated a violation of behavioral norms. For example: “I understand you’re angry, but you shouldn’t use language like this. Twitter is a much nicer place when we respectfully tweet our opinions.”

The intervention involved delivering a direct and publicly visible counterspeech response to the xenophobic tweet, within 24 hours of the subject’s initial posting. To administer the treatments, we utilized six human-controlled sockpuppet accounts created at least four weeks prior to the start of the field phase. The sockpuppets’ profile pictures depicted distant silhouettes of humans, as seen from behind, conveying no gender, race, ethnicity, or nationality information. These accounts maintained an apolitical posting history. To maintain authenticity, we interspersed the counterspeech posts with neutral messages and images. At the beginning of the field phase, for each sockpuppet account we bought approximately 900 followers and 200–300 friends.

### Outcomes

We collected three preregistered primary outcomes 28 days after applying the treatment. *Xenophobic Tweet Deleted* measures whether the sender deleted their xenophobic tweet. *No. of Xenophobic Tweets* measures the (absolute) number of xenophobic tweets posted in the four weeks after the intervention. We define as xenophobic any tweet that (i) includes at least one keyword from our dictionary and (ii) for which the Perspective API predicts a probability of at least 50% to contain severe toxicity^27,28^. This outcome variable is not defined for 8 users whose post-treatment tweets could not be classified as they only contained links or text in non-English languages. *Xenophobic Tweet Share* measures the share of xenophobic tweets over the total number of tweets posted in the four weeks after the intervention. This outcome is not defined for an additional 46 users who did not post anything after the intervention. To test for the silencing effect found in a previous study^19^, the SI provides results for a secondary preregistered outcome: the total number of tweets sent during the four-week follow-up period. Summary statistics for all outcomes are available in Table s7. Further, we collect two measures of engagement with the original hate tweet: the *No. of likes received by xenophobic tweet* as a measure of affirmation of the original hate tweet, and the *No. of retweets of xenophobic tweet* to capture propagation.

### Statistical analysis

This study is affected by two sources of attrition. The first source consists of a small number of users whose xenophobic tweet was deleted in the short time window between the eligibility checks and treatment application. We conducted the same check for the control group and excluded all users who deleted their tweet before treatment would have been applied. Table s6 provides balance tests confirming that, as expected by randomization, the pre-treatment characteristics of the experimental subjects are indeed balanced across experimental conditions.

The second source of attrition concerns users who deleted their accounts (N = 61), set them to private (N = 12), or were suspended by Twitter (N = 253) between the treatment application and the end of the four-week study period. We were not able to retrieve outcome data for an additional 13 accounts. We exclude these accounts from the analysis. Reassuringly, we find little evidence that the counterspeech treatments predict attrition. The share of users who deleted their account or set it to private, i.e., performed actions to make their tweets invisible to the public, is similar across experimental conditions. Relative to the control group, the difference in this share of users is $\beta =0.006$, $p=0.572$ for disapproval; $\beta =0.008$, $p= 0.434$ for traditional perspective-taking; $\beta =0.012$, $p=0.289$ for analogical perspective-taking; and $\beta =-0.003$, $p=0.721$ for perspective getting. This analysis yields similar results if we focus on all attrited users (including those suspended by Twitter). The only exception is that attrition is slightly lower for users in the perspective-getting treatment compared to the control group ($\beta =-0.041$, $p=0.029$). Table s4 reports the full analysis results. Table s5 shows that the treatment effect estimates are robust to including users who are subject to attrition, after re-coding the values of their outcome variables to a constant.

The remaining analysis sample contains $N=2102$ users, of which $N=337$ are assigned to traditional perspective-taking, $N=357$ to analogical perspective-taking, $N=399$ to perspective-getting, $N=377$ to disapproval, and $N=632$ to the control group. For each experimental condition, we report the mean of the outcome variable along with the 95% confidence interval. We also report the treatment effect estimated from regressing the outcome on the assigned experimental condition, without adjusting for additional covariates. We provide standard errors, two-sided p-values, and Benjamini–Hochberg adjusted p-values to account for multiple hypothesis testing; the latter are shown only for pre-registered analyses. When analyzing the differences across treatment arms, the smaller sample size limits the power of the analysis. To partially account for this, we deviate from our preregistered specification to include controls selected with the post-double-Lasso method^29^. This procedure adjusts for possible imbalances in pre-treatment covariates and variance reduction. We indicate coefficients estimated with this procedure as $\beta _L$. Tables s1 and s2 report the complete estimates for all regressions, both with and without covariates, following our preregistered analyses.

## Supplementary Information

Supplementary Information.

## Data Availability

Replication materials, including anonymized data and code, are available at the Harvard Dataverse (https://doi.org/10.7910/DVN/LNWU6W).
