# Supplementary material for: Counterspeech encouraging users to adopt the perspective of minority groups reduces hate speech and its amplification on social media
Source: Sci Rep. 2025 Jul 1;15:22018. doi: 10.1038/s41598-025-05041-w (PMC12215266; doi:10.1038/s41598-025-05041-w)
Supplement: Supplementary file 1 — Supplementary Information. [file 41598_2025_5041_MOESM1_ESM.pdf]

Supplementary Information

for

Counterspeech encouraging users to adopt the perspective of minority groups reduces hate speech and its amplification on social media

1 Main Results

1.1 Ethical considerations

This study has been approved without reservation by [*institution and protocol reference to be added upon acceptance*]. In this section, we report the main ethical considerations discussed in the application and subsequent approval.

Participants' consent. The subjects of this study do not give their informed consent to participate in the experiment. Two main reasons guide this decision. First, obtaining their informed consent could affect their behavior and introduce experimenter effects, whereby the participants alter their natural behavior because they know they are being observed. Second, this would also expose researchers' identities to mostly anonymous online users. As some of the participants may be active members of online hate groups, we are concerned about potential online and offline consequences for researchers' safety, including bullying and harassment. Our approach is in line with similar studies conducting digital field experiments on Twitter[1,2] and respects contemporary Twitter's policy regarding academic research. Moreover, Twitter users are aware that their posts are public, and receiving replies, including counterspeech, is a usual aspect of the platform experience. Users have the option of restricting the public availability of their posts if they wish (by making their accounts private).

Backlash. Another concern is that counterspeech messages may backfire, producing an increase in the use of xenophobic hate speech rather than a reduction. We have selected non-hostile strategies that are theorized to be effective; therefore, we do not expect a significant backlash effect[3]. Previous studies have documented only short-lived[4] or no backlash[2,5]. Our expectation was that the minimal risk of a backlash would be offset by the reduction in hate speech in the medium and long term. These expectations were ultimately confirmed by the results of the study.

Participants' identity. Subjects could be exposed to a data protection risk if their Twitter handle, User ID, or the content of their tweets were revealed. To mitigate these risks during the project phase, we stored the data in three tiers. First, each subject received a randomly generated pseudonym number. Personal identifiers were stored alongside the corresponding pseudonym numbers in an identification key data set, separate from other data. Second, the tweets were stored independently and identified only by the pseudonym numbers. In a third dataset, their post-treatment behavior was sorted using the pseudonym numbers. Throughout the project, data access was limited to the research team and safeguarded by the [*institution and protocol reference to be added upon acceptance*] IT infrastructure under an identity management system. All members of the research team were informed of the [*institution and protocol reference to be added upon acceptance*] research code of conduct. Upon publication, the text of the tweets and all identifying information will be deleted. The pseudo-anonymized data will be published in a public repository for the purpose of scientific replication.

1.2 Main regression table

Figure 2 in the main paper reports the mean for each outcome of interest and its confidence interval across treatment arms. In Panel 1 of Table s1, we follow our preregistered analysis and report the treatment effects obtained by regressing each of our outcomes on dummy variables that take the value of 1 for each treatment group and 0 for the control. To ease interpretation, we standardize each outcome by subtracting the mean and dividing by the standard deviation. The table includes all four preregistered outcomes (*Xenophobic Tweet Deleted, No. Xenophobic Tweets, Xenophobic Tweet Share, No. Total Tweets*).

The regression estimates confirm the results presented in the paper. While counterspeech based on disapproval seems to increase the probability of deleting the original xenophobic tweet and decrease the creation of new xenophobic speech, the estimated treatment effects are small in magnitude and do not reach conventional levels of statistical significance. On the other hand, all counterspeech interventions based on perspective appear to have larger and more precisely estimated treatment effects.

Panel 2 in Table s1 reports the main results after including controls. The controls have been specified in the preregistered analysis and include: number of days since account creation (in quintiles), the number of xenophobic tweets in the month before the intervention, the total number of tweets in the month before the intervention, the number of followers and the number

of friends at the time of the intervention, and the average tweet length at the time of the intervention. Results are virtually unchanged after the inclusion of controls.

Panel 3 in Table s1 reports the main results after including preregistered controls and fixed effects for treatment weeks.

Columns 3 and 4, 7 and 8, and 11 and 12 report a smaller number of observations. This is due to the fact that those outcome variables are only defined if two conditions are met: (i) the post-treatment timeline is not empty, and (ii) at least one of the post-treatment tweets can be processed by our classifier. For instance, tweets that only include links or are written in a language other than English cannot be classified. Similarly, some of the control variables are not specified for all users, resulting in an additional, albeit minor, drop in the number of observations in columns 7 and 8.

We use Seemingly Unrelated Regression to test the joint null hypotheses of a non-significant effect of each treatment across all outcome variables. To this end, we fit a system of equations in which all four preregistered outcomes are regressed on a single treatment indicator. A two-sided test of the joint significance of all coefficients across regressions is used to reject the null hypothesis. Results are reported in the main paper and indicate that the null hypothesis can only be rejected for the treatment based on analogical perspective-taking and for all perspective treatments combined.

1.3 Engagement Results

For each outcome, Figure 3 in the main paper reports its mean and confidence interval across treatment arms. Table s2 reports the results of regressing each measure of engagement on treatment indicators. The Table includes only results for outcomes that are equally well-defined for each treatment group and the control group. In particular, we estimate the effect of our counterspeech interventions on the number of likes and the number of retweets received by the original xenophobic tweet, but not on the number of replies to the counterspeech tweet (which is not defined for the control group).

Columns 1 to 6 report the estimates of a regression of the outcome variables on treatment indicators. Columns 7 to 12 present the same results after applying the inverse hyperbolic sine transformation to the outcome variables. In all columns, the outcome variables are standardized by subtracting the mean and dividing by the standard deviation. In Panel 1, we report the treatment effects obtained by regressing each of our outcomes on dummy variables that take the value of 1 for each treatment group and 0 for the control group, without additional control variables. In Panel 2 we include preregistered controls. In Panel 3, we further add fixed effects for treatment weeks.

The results confirm that perspective-centered interventions reduce engagement with the xenophobic tweet and, in particular, decrease the number of likes and retweets obtained four weeks after the intervention. While the effect of disapproval-based intervention goes in the same direction, the estimated effects are small in size and not statistically significant.

We use Seemingly Unrelated Regression to test the joint-null hypotheses of a non-significant effect of each treatment across all outcome variables. Results are reported in the main paper and indicate that the null hypothesis can only be rejected for the treatment based on analogical perspective-taking, as well as for all perspective treatments combined.

1.4 Descriptive results on engagement with the counterspeech tweet

Figure s1 reports descriptive results on two additional measures of engagement: the number of likes and the number of retweets received by the counterspeech tweet. As the control group does not receive any intervention, these findings have only descriptive value. This analysis shows that treatment strategies differ in the extent to which the counterspeech tweet receives support from bystanders. In particular, disapproval counterspeech tweets receive the highest number of likes (*No. of likes received by counterspeech tweet*, not preregistered). However, the second panel suggests that none of the counterspeech messages obtain a number of retweets that is significantly different from zero (*No. of retweets of counterspeech tweet*, not preregistered). Overall, this descriptive analysis does not indicate the existence of clear patterns of engagement with the counterspeech tweet.

1.5 Effects of perspective-centered treatments against disapproval

Table s3 reports the effects of individual perspective-based treatments compared to the disapproval treatment condition.

2 Deviations from the Pre-Analysis Plan

Hypotheses. We preregistered the following hypotheses: **H1**: Disapproval has an effect on reducing xenophobic hate speech compared to the control group.
H2: Perspective-centered treatments (i.e., perspective-getting, traditional perspective-taking or analogical perspective-taking, and zero otherwise) have a stronger effect in reducing xenophobic hate speech than disapproval.
H2a: Perspective-getting will have the strongest effect among all treatments in reducing xenophobic hate speech.
H2b: Traditional perspective-taking will have a stronger effect than analogical perspective-taking, but a weaker effect than perspective getting.
H3: Perspective-centered treatments (i.e., perspective-getting, traditional perspective-taking and analogical perspective-taking) have more durable effects in reducing xenophobic hate speech than disapproval.

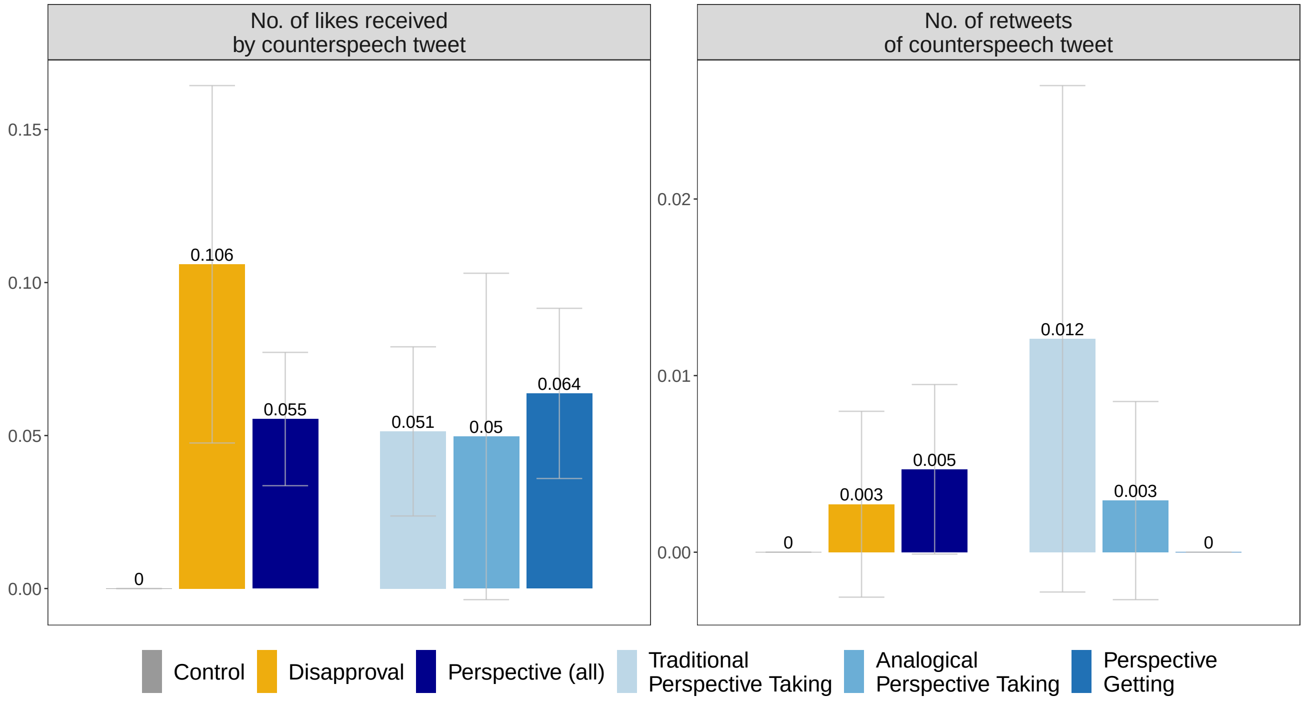

Figure s1. Vertical bars are group-level averages along 90% and 95% confidence intervals measuring the number of likes received by the counterspeech tweet (*No. of likes received by counterspeech tweet*), and the number of retweets of the counterspeech tweet (*No. of retweets of counterspeech tweet*), across the different treatment and control groups over the 4-week follow-up period.

We do not find support for Hypothesis 1. While the estimated effects are all in the expected direction, they are not statistically significant. We find limited evidence that the perspective-centered treatments are stronger than the disapproval treatment (Hypothesis 2): compared to the control group, we find support indicating that perspective-centered treatments have an effect on the deletion of the original hate tweet. Finally, contrary to Hypotheses 2a-b, we find suggestive evidence that analogical perspective-taking has the strongest effects on outcomes, even though those results should be taken with caution due to the limited power to detect subgroup effects.

The preregistration primarily framed the hypotheses in terms of the relative effectiveness of different treatment arms (e.g. ”Perspective-getting will have the strongest effect among all treatments in reducing racist hate speech”). However, our analysis indicates that we lack the statistical power to draw definitive conclusions about relative effectiveness. As a result, the study focuses more extensively on the effectiveness of individual treatment arms compared to the control group, while also reporting relative effectiveness with appropriate caution.

Hypothesis 3, which aimed to compare the long-term effectiveness of perspective-centered treatments with the disapproval treatment, is particularly affected by this limitation. Our analysis shows null effects. These results are available upon request from the corresponding author but are not included in the study, as any conclusions drawn would be speculative.

Data collection and Sampling. The data collection, randomization, and treatment assignment took place as preregistered. Compared to the preregistration, we extended the data collection period due to slower sampling than expected. We preregistered the collection of a sample of 2460 xenophobic tweets from November 17, 2021, until approximately January 15, 2022, or until the target sample size of 2460 was met. Due to much slower sampling rates than anticipated, the research team had to decide on January 15, 2022, between working with a significantly smaller sample or extending the sampling for two more weeks. We opted for the latter to avoid being severely underpowered for the study and continued the sampling process until January 30, 2022, at which point we had 2441 participants and had nearly reached the preregistered sample size of 2460.

Outcomes. We preregistered three primary outcomes: (1) a binary indicator of whether the original xenophobic tweet was deleted 4 weeks after the intervention (*Xenophobic Tweet Deleted*), (2) the absolute number of xenophobic hate speech tweets posted during the 4-week period following the treatment (*No. Xenophobic Tweets*), and (3) the ratio of xenophobic hate speech to all tweets in the 4-week period after the treatment (*Xenophobic Tweet share*). We analyze and report the findings for these outcomes as preregistered.

A fourth outcome–the total number of tweets sent by an account– was preregistered to help distinguish general silencing effects from specific reductions in hate speech. However, as specified in the preregistration, this outcome is not derived from our main hypotheses and is treated as a supplementary measure. We report results for this outcome in the SI (*No. Total Tweets*).

Moreover, we preregistered to measure the amplification of the xenophobic tweets that constitute our main sample. As planned, we collect data on likes (*No. of likes received by the xenophobic tweet*) and, additionally, on retweets (*No. of retweets of xenophobic tweet*, not preregistered). The analysis of the treatment effect on amplification was preregistered as supplementary; however, we report it in the main text.

Finally, we preregistered the collection of replies to the treatment tweet (*No. of user's replies to counterspeech tweet*). In an exploratory analysis, we annotate these replies and report in the main text on how often treated users apologize, double down or attack the sock puppet account, or respond neutrally.

Data Analysis. We preregistered two model specifications: unconditional OLS of the outcomes on treatment indicators and conditional OLS with preregistered controls. In the paper, we report results from the first specification. Conditional effects are reported only in the SI, as they do not differ from the unconditional effects.

Further, we conduct an exploratory analysis of the effects of perspective-centered treatments compared to the disapproval condition. In this case, we present results from a non-preregistered model in which controls are selected using the post-double-Lasso method[6]. This approach adjusts for potential imbalances in pre-treatment covariates, reduces variance, and alleviates concerns related to small sample sizes and limited power.

Robustness. Attrition occurs when users delete their accounts, set them to private, or are suspended by Twitter. We follow our preregistered analysis and test for differential user-driven or platform-driven attrition between the treatment arms and the control group. Because outcome data could not be retrieved for 13 accounts due to unknown technical issues, we extend the attrition analysis to include these cases. In addition, we account for the possibility of false discoveries by reporting Benjamini-Hochberg adjusted p-values and results from a Seemingly Unrelated Regression model, as defined in our preregistration.

3 Robustness

3.1 Attrition

Table s4 reports results on attrition. In particular, we regress a dummy indicator for attrition on treatment indicators. Each coefficient can be interpreted as the difference in attrition between the treatment and control groups. Following our preregistered analysis, we separate different sources of attrition. Specifically, we identify cases where attrition is due to users deleting their accounts or setting them to private. These actions could potentially occur in response to our treatment. Column 1 shows that this is not the case: users' decisions regarding their accounts are balanced across the treatment and control groups. Column 2 shows that users in the perspective-getting group are slightly less likely to be suspended compared to users in the control group. Columns 3 and 4 show the aggregate imbalances in compound sources of attrition across treatment arms.

To gauge the possible size of the bias induced by attrition, we estimate the treatment effect after recoding the outcome variables for attriters. In this case, instead of dropping them from the sample, the users who are subject to attrition receive a value of 1 for the outcome variable *Xenophobic Tweet deleted*, and 0 for *No. Total Tweets*, *No. Xenophobic Tweets*, and *Xenophobic Tweet Share*. Results are reported in Table s5. The main findings do not appear to be affected by attrition.

3.2 Balance of Pre-Treatment Characteristics

Table s6 reports the results of regressing each pre-treatment variable on treatment indicators. These variables have been identified as suitable controls in the preregistered report. Only one of the 24 coefficients is statistically significant at a 95% level.

4 Additional information

4.1 Summary Statistics

Table s7 reports the main summary statistics of all outcome variables used in this analysis. These include the number of non-missing observations, the mean, standard deviation, and median of each variable, along with the minimum and maximum values observed in the sample.

4.2 Dictionary

The following terms were used to search the Twittersphere for potentially xenophobic tweets. The dictionary includes xenophobic terms, racial slurs, and neutral terms referring to commonly targeted groups. Xenophobic terms and racial slurs were drawn from the HateBase database and have been selected because they are classified as extremely offensive and are sufficiently

common (more than 100 reported sightings).

Africoon, Africoons, Anchor Baby, Beaner Baby, Beaners, Blacks, Bluegum, Bogan, Bogans, Bohunk, Border Bunny, Border Hopper, Border Jumper, Border Jumpers, Brown Invaders, Camel Fucker, Camel Fuckers, Camel Humper, Camel Humpers, Camel Jockes, Chinaman, Chinamen, Ching Chong, Ching Chongs, Chink, Chinks, Coloreds, Congoid, Congoids, Coon, Coon ass, Cotton picker, Cotton pickers, Golliwog, Golliwogs, Gooks, Gooky, Groid, Groids, Halfricans, Hebro, Heeb, Heebs, Holoax, Hymie, Immigrant, Immigrants, Injuns, Jew, Jews, Jigaboo, Jigaboos, Jiggaboo, Jiggaboos, Jungle bunny, Kike, Kikes, Kneegrow, Migrant, Migrants, Mockey, Moon cricket, Moshead, Moulie, Moulinyan, Mud Duck, Mud Ducks, Mud Shark, Mulato, Mulatto, Mulsa, Muslamic, Muslim, Muslims, Negro, Negroes, Nicca, Niccas, Nig, Nig nog, Nig nogs, Nigger, Niggers, Nigette, Nigettes, Nigglet, Nigglets, Nigglette, Niggress, Nips, Pavement Ape, Piccaninnies, Pickaninny, Porch Monkey, Porch Monkeys, Quadroon, Race Traitors, Raghead, Refugee, Refugees, Sambos, Sand Monkey, Sand Monkeys, Sand Nigger, Sooty, Spear Chucker, Spic, Tar Baby, Tar Babies, Towel Head, Towel Heads, Uncle Tom, Wagon Burner, Wetback, White Genocide, Yard Ape, Yids, Zionazi, Zipperhead

4.3 Geolocation

To determine user location, we relied on self-reported location information available in the user's profile and cross-referenced it with the GeoNames database to match locations with countries. In cases where no location information was provided, we assigned users the most common location among their followers, as done in previous work[7].

References

1. Munger, K. Tweetment effects on the tweeted: Experimentally reducing racist harassment. *Polit. Behav.* **39**, 629–649 (2017).
2. Hangartner, D. *et al.* Empathy-based counterspeech can reduce racist hate speech in a social media field experiment. *Proc. Natl. Acad. Sci.* **118**, e2116310118 (2021).
3. Benesch, S., Ruths, D., Dillon, K. P., Saleem, H. M. & Wright, L. Considerations for successful counterspeech. Dangerous Speech Project (2016).
4. Munger, K. Don't@ me: Experimentally reducing partisan incivility on twitter. *J. Exp. Polit. Sci.* **8**, 102–116 (2021).
5. Guess, A. & Coppock, A. Does counter-attitudinal information cause backlash? results from three large survey experiments. *Br. J. Polit. Sci.* **50**, 1497–1515 (2020).
6. Belloni, A., Chernozhukov, V. & Hansen, C. Inference on treatment effects after selection among high-dimensional controls. *The Rev. Econ. Stud.* **81**, 608–650 (2014).
7. Barberá, P. *et al.* Who leads? who follows? measuring issue attention and agenda setting by legislators and the mass public using social media data. *Am. Polit. Sci. Rev.* **113**, 883–901 (2019).

Table s1. Treatment effect estimates

	OLS results				OLS results with pre-registered controls				OLS results with pre-registered controls and week fixed-effects			
	Xenophobic Tweet Deleted (1)	No. Total Tweets (2)	No. Xenophobic Tweets (3)	Xenophobic Tweet Share (4)	Xenophobic Tweet Deleted (5)	No. Total Tweets (6)	No. Xenophobic Tweets (7)	Xenophobic Tweet Share (8)	Xenophobic Tweet Deleted (9)	No. Total Tweets (10)	No. Xenophobic Tweets (11)	Xenophobic Tweet Share (12)
Disapproval												
β	0.061	0.032	-0.033	-0.105*	0.055	0.077*	-0.057	-0.131*	0.056	0.078*	-0.052	-0.127*
SE	[0.060]	[0.065]	[0.068]	[0.062]	[0.061]	[0.040]	[0.064]	[0.067]	[0.061]	[0.041]	[0.065]	[0.067]
Obs	1009	1009	1007	995	1003	1003	1001	989	1003	1003	1001	989
Perspective: All treatments combined												
β	0.14***	-0.002	-0.035	-0.084	0.141***	0.036	-0.015	-0.086	0.137***	0.036	-0.013	-0.090
SE	[0.047]	[0.049]	[0.056]	[0.062]	[0.046]	[0.029]	[0.049]	[0.062]	[0.047]	[0.030]	[0.049]	[0.063]
Obs	1725	1725	1717	1692	1713	1713	1705	1680	1713	1713	1705	1680
Traditional Perspective Taking												
β	0.125*	-0.011	-0.037	-0.053	0.134**	0.004	-0.007	-0.041	0.133**	0.004	-0.004	-0.041
SE	[0.067]	[0.066]	[0.065]	[0.089]	[0.067]	[0.04]	[0.057]	[0.086]	[0.067]	[0.041]	[0.057]	[0.086]
Obs	969	969	963	950	960	960	954	941	960	960	954	941
Analogical Perspective Taking												
β	0.172**	0.026	-0.053	-0.113*	0.178***	0.056	-0.035	-0.111*	0.174**	0.055	-0.032	-0.111*
SE	[0.069]	[0.067]	[0.065]	[0.061]	[0.068]	[0.041]	[0.061]	[0.061]	[0.068]	[0.041]	[0.061]	[0.063]
Obs	989	989	987	972	983	983	981	966	983	983	981	966
Perspective Getting												
β	0.124*	-0.019	-0.015	-0.084	0.118*	0.043	0.005	-0.095	0.109*	0.043	0.004	-0.102
SE	[0.063]	[0.064]	[0.070]	[0.066]	[0.062]	[0.039]	[0.063]	[0.069]	[0.063]	[0.039]	[0.063]	[0.071]
Obs	1031	1031	1027	1012	1024	1024	1020	1005	1024	1024	1020	1005

Notes. Notes. Each β coefficient shows the result of regressing each outcome variable on a binary treatment indicator that takes the value 1 for units in the treatment group, and 0 for units in the control group. Controls are: quintiles of account age, number of xenophobic tweets in the month before the intervention, total number of tweets in the month before the intervention, number of followers, number of friends, average tweet length before the intervention. *SE* indicate robust standard errors. *,**, *** denote significance at the 10%, 5%, and 1% levels, respectively.

Table s2. Engagement results

	OLS results		OLS results with pre-registered controls		OLS results with pre-registered controls and week fixed-effects		OLS results		OLS results with pre-registered controls		OLS results with pre-registered controls and week fixed-effects	
	No. of likes received by xenophobic tweet	No. of retweets of xenophobic tweet	No. of likes received by xenophobic tweet	No. of retweets of xenophobic tweet	No. of likes received by xenophobic tweet	No. of retweets of xenophobic tweet	No. of likes received by xenophobic tweet (i.h.s)	No. of retweets of xenophobic tweet (i.h.s)	No. of likes received by xenophobic tweet (i.h.s)	No. of retweets of xenophobic tweet (i.h.s)	No. of likes received by xenophobic tweet (i.h.s)	No. of retweets of xenophobic tweet (i.h.s)
	(1)	(2)	(3)	(4)	(5)	(6)	(7)	(8)	(9)	(10)	(11)	(12)
Disapproval												
β	-0.059	-0.094	-0.014	-0.055	-0.013	-0.051	-0.035	-0.058	-0.015	-0.040	-0.014	-0.038
SE	[0.082]	[0.067]	[0.069]	[0.044]	[0.067]	[0.042]	[0.070]	[0.064]	[0.070]	[0.060]	[0.070]	[0.062]
Obs	1003	1003	997	997	997	997	1003	1003	997	997	997	997
Perspective (all)												
β	-0.133**	-0.073	-0.101**	-0.037	-0.099**	-0.032	-0.093*	-0.035	-0.066	-0.001	-0.070	0.001
SE	[0.064]	[0.068]	[0.049]	[0.045]	[0.047]	[0.043]	[0.052]	[0.055]	[0.050]	[0.048]	[0.050]	[0.048]
Obs	1710	1710	1698	1698	1698	1698	1710	1710	1698	1698	1698	1698
Traditional Perspective Taking												
β	-0.114*	0.016	-0.058	0.068	-0.055	0.076	-0.091	0.124	-0.063	0.148*	-0.065	0.151*
SE	[0.068]	[0.086]	[0.047]	[0.061]	[0.047]	[0.061]	[0.069]	[0.088]	[0.068]	[0.079]	[0.068]	[0.080]
Obs	962	962	953	953	953	953	962	962	953	953	953	953
Analogical Perspective Taking												
β	-0.150**	-0.106	-0.116**	-0.057	-0.113**	-0.055	-0.113*	-0.067	-0.082	-0.021	-0.086	-0.018
SE	[0.065]	[0.066]	[0.050]	[0.043]	[0.049]	[0.043]	[0.063]	[0.060]	[0.063]	[0.056]	[0.063]	[0.055]
Obs	980	980	974	974	974	974	980	980	974	974	974	974
Perspective Getting												
β	-0.132**	-0.118*	-0.106*	-0.089	-0.100*	-0.079	-0.078	-0.140**	-0.049	-0.107**	-0.055	-0.103**
SE	[0.065]	[0.066]	[0.055]	[0.055]	[0.051]	[0.049]	[0.063]	[0.057]	[0.061]	[0.050]	[0.062]	[0.050]
Obs	1024	1024	1017	1017	1017	1017	1024	1024	1017	1017	1017	1017

Notes. Each β coefficient shows the result of regressing each outcome variable on a binary treatment indicator that takes the value 1 for units in the treatment group, and 0 for units in the control group. Controls are: quintiles of account age, number of xenophobic tweets in the month before the intervention, total number of tweets in the month before the intervention, number of followers, number of friends, average tweet length before the intervention. *SE* indicate robust standard errors. *,**, *** denote significance at the 10%, 5%, and 1% levels, respectively.

Table s3. Effects against the disapproval condition

	Xenophobic Tweet Deleted	No. Total Tweets	No. Xenophobic Tweets	Xenophobic Tweet Share	No. of likes received by xenophobic tweet	No. of retweets of xenophobic tweet	No. of replies received by counterspeech tweet	No. of negative replies received by counterspeech tweet
	(1)	(2)	(3)	(4)	(5)	(6)	(7)	(8)
Empathy: All treatments combined								
β	0.079	-0.040	0.010	0.025	-0.070	0.018	0.035	-0.048
	(0.061)	(0.038)	(0.052)	(0.037)	(0.054)	(0.024)	(0.074)	(0.077)
Obs	1470	1470	1464	1445	1337	1337	1266	1266
Empathy: Traditional Perspective Taking								
β	0.068	-0.064	0.005	0.052	-0.053	0.098*	0.064	-0.045
	(0.077)	(0.048)	(0.063)	(0.073)	(0.059)	(0.058)	(0.095)	(0.095)
Obs	714	714	710	703	656	656	615	615
Empathy: Analogical Perspective Taking								
β	0.133*	-0.009	0.013	0.013	-0.092*	-0.012	-0.145*	-0.083
	(0.081)	(0.048)	(0.057)	(0.033)	(0.054)	(0.018)	(0.086)	(0.093)
Obs	734	734	734	725	668	668	645	645
Empathy: Perspective Getting								
β	0.071	-0.035	0.036	0.028	-0.073	-0.030*	0.159*	0.020
SE	(0.076)	(0.045)	(0.066)	(0.041)	(0.056)	(0.016)	(0.091)	(0.094)
Obs	776	776	774	765	713	713	668	668

Notes. Each β coefficient shows the result of regressing each outcome variable on a binary treatment indicator that takes the value 1 for the perspective-based treatment group, and 0 for disapproval (control observations are dropped). Regression models include pre-treatment controls selected using Lasso-based post-double selection. Robust standard errors are in parenthesis. *,**, *** denote significance at the 10%, 5%, and 1% levels, respectively.

Table s4. Attrition

	(1) Deleted, Private	(2) Suspended	(3) Deleted, Private + Suspended	(4) Deleted, Private + Suspended + Human Error
Perspective (all)	0.005	0.005	0.010	0.006
	(0.008)	(0.014)	(0.016)	(0.016)
Disapproval	0.006	0.007	0.013	0.009
	(0.01)	(0.018)	(0.021)	(0.021)
Observations	2441	2441	2441	2441
R-squared	0.000	0.000	0.000	0.000
Traditional	0.008	0.028	0.037*	0.036
	(0.011)	(0.020)	(0.022)	(0.023)
Analogical	0.012	0.020	0.032	0.026
	(0.011)	(0.019)	(0.022)	(0.022)
Perspective Getting	-0.003	-0.032*	-0.035*	-0.041**
	(0.009)	(0.016)	(0.018)	(0.019)
Disapproval	0.006	0.007	0.013	0.009
	(0.01)	(0.018)	(0.021)	(0.021)
Observations	2441	2441	2441	2441
R-squared	0.001	0.004	0.005	0.005

*Notes.*Each β coefficient shows the result of regressing each outcome variable on a binary treatment indicator. The dependent variables sum different sources of attrition. Robust standard errors are in parenthesis. *,**, *** denote significance at the 10%, 5%, and 1% levels, respectively.

Table s5. Treatment effect estimates, including attriters

	Xenophobic Tweet Deleted (1)	No. Total Tweets (2)	No. Xenophobic Tweets (3)	Xenophobic Tweet Share (4)
Disapproval				
β	0.055	0.020	-0.033	-0.099*
SE	(0.059)	(0.060)	(0.063)	(0.058)
Obs	1170	1170	1168	1156
Perspective: All treatments combined				
β	0.092**	-0.007	-0.034	-0.079
	(0.046)	(0.045)	(0.052)	(0.058)
Obs	2001	2001	1993	1968
Traditional Perspective Taking				
β	0.152**	-0.040	-0.045	-0.056
	(0.064)	(0.060)	(0.060)	(0.081)
Obs	1136	1136	1130	1117
Analogical Perspective Taking				
β	0.154**	0.000	-0.056	-0.108*
	(0.063)	(0.062)	(0.060)	(0.057)
Obs	1155	1155	1153	1138
Perspective Getting				
β	-0.025	0.017	-0.001	-0.072
	(0.057)	(0.061)	(0.067)	(0.062)
Obs	1170	1170	1166	1151

Notes. Each β coefficient shows the result of regressing each outcome variable on a binary treatment indicator that takes the value 1 for units in the treatment group, and 0 for units in the control group. The sample include users subject to attrition. For them, *Xenophobic Tweet Deleted* is re-coded to 1, all other outcomes are re-coded to 0. Robust standard errors are in parenthesis. *,**, *** denote significance at the 10%, 5%, and 1% levels, respectively.

Table s6. Balance

	Outcome Variable	Treatment	β	SE	Pval
1	Account Age	Traditional Perspective Taking	-0.088	0.062	0.161
2	Account Age	Analogical Perspective Taking	-0.026	0.062	0.672
3	Account Age	Perspective Getting	-0.097	0.059	0.102
4	Account Age	Disapproval	-0.092	0.061	0.130
5	Followers Count	Traditional Perspective Taking	-0.122	0.061	0.046
6	Followers Count	Analogical Perspective Taking	-0.104	0.066	0.114
7	Followers Count	Perspective Getting	-0.070	0.069	0.308
8	Followers Count	Disapproval	-0.099	0.064	0.122
9	Avg Length of Tweets	Traditional Perspective Taking	0.096	0.064	0.136
10	Avg Length of Tweets	Analogical Perspective Taking	0.048	0.060	0.423
11	Avg Length of Tweets	Perspective Getting	0.064	0.060	0.280
12	Avg Length of Tweets	Disapproval	0.000	0.059	0.997
13	No of Tweets	Traditional Perspective Taking	-0.005	0.064	0.939
14	No of Tweets	Analogical Perspective Taking	-0.040	0.063	0.521
15	No of Tweets	Perspective Getting	-0.062	0.059	0.294
16	No of Tweets	Disapproval	-0.060	0.060	0.316
17	No of Xenophobic Tweets	Traditional Perspective Taking	-0.064	0.063	0.309
18	No of Xenophobic Tweets	Analogical Perspective Taking	-0.045	0.071	0.525
19	No of Xenophobic Tweets	Perspective Getting	-0.067	0.065	0.302
20	No of Xenophobic Tweets	Disapproval	0.005	0.069	0.945
21	Friends Count	Traditional Perspective Taking	0.002	0.066	0.979
22	Friends Count	Analogical Perspective Taking	-0.060	0.055	0.275
23	Friends Count	Perspective Getting	-0.056	0.053	0.292
24	Friends Count	Disapproval	0.055	0.074	0.453

*Notes.*Each β coefficient shows the result of regressing each outcome variable on a binary treatment indicator. Robust standard errors and P-values are reported.

Table s7. Summary Statistics

	N	Mean	SD	Median	Min	Max
Xenophobic Tweet Deleted	2102	0.072	0.259	0.000	0.000	1.000
No. of Total Tweets	2102	504.061	598.118	274.000	0.000	3199.000
No. of Xenophobic Tweets	2094	0.831	2.933	0.000	0.000	48.000
Xenophobic Tweet Share	2066	0.004	0.024	0.000	0.000	0.667
No. of likes received by xenophobic tweet	1933	1.442	8.324	0.000	0.000	250.000
No. of retweets of xenophobic tweet	1933	0.189	1.956	0.000	0.000	73.000
No. of replies to xenophobic tweet	2102	0.200	0.440	0.000	0.000	3.000
No. of likes received by counterspeech tweet	1433	0.068	0.432	0.000	0.000	9.000
No. of retweets of counterspeech tweet	1433	0.004	0.075	0.000	0.000	2.000

*Notes.*Summary Statistics of the main outcome variables.
